# Changes in Cancer Mortality by Race and Ethnicity Following the Implementation of the Affordable Care Act in California

**DOI:** 10.3389/fonc.2022.916167

**Published:** 2022-07-13

**Authors:** Maria Elena Martinez, Scarlett L. Gomez, Alison J. Canchola, Debora L. Oh, James D. Murphy, Winta Mehtsun, K. Robin Yabroff, Matthew P. Banegas

**Affiliations:** ^1^ Herbert Wertheim School of Public Health and Human Longevity Science, University of California, San Diego, La Jolla, CA, United States; ^2^ Moores Cancer Center, University of California, San Diego, La Jolla, CA, United States; ^3^ Department of Epidemiology and Biostatistics, University of California, San Francisco, San Francisco, CA, United States; ^4^ Helen Diller Family Comprehensive Cancer Center, University of California, San Francisco, CA, United States; ^5^ Department of Radiation Medicine and Applied Sciences, University of California, San Diego School of Medicine, La Jolla, CA, United States; ^6^ Department of Surgery, University of California, San Diego School of Medicine, La Jolla, CA, United States; ^7^ Surveillance and Health Equity Science, American Cancer Society, Kennesaw, GA, United States

**Keywords:** Affordable Care Act, race and ethnicity, disparities, breast cancer, cervix cancer, colorectal cancer

## Abstract

Although Affordable Care Act (ACA) implementation has improved cancer outcomes, less is known about how much the improvement applies to different racial and ethnic populations. We examined changes in health insurance coverage and cancer-specific mortality rates by race/ethnicity pre- and post-ACA. We identified newly diagnosed breast (n = 117,738), colorectal (n = 38,334), and cervical cancer (n = 11,109) patients < 65 years in California 2007-2017. Hazard rate ratios (HRR) and 95% confidence intervals (CI) were calculated using multivariable Cox regression to estimate risk of cancer-specific death pre- (2007-2010) and post-ACA (2014-2017) and by race/ethnicity [American Indian/Alaska Natives (AIAN); Asian American; Hispanic; Native Hawaiian or Pacific Islander (NHPI); non-Hispanic Black (NHB); non-Hispanic white (NHW)]. Cancer-specific mortality from colorectal cancer was lower post-ACA among Hispanic (HRR = 0.82, 95% CI = 0.74 to 0.92), NHB (HRR = 0.69, 95% CI = 0.58 to 0.82), and NHW (HRR = 0.90; 95% CI = 0.84 to 0.97) but not Asian American (HRR = 0.95, 95% CI = 0.82 to 1.10) patients. We observed a lower risk of death from cervical cancer post-ACA among NHB women (HRR = 0.68, 95% CI = 0.47 to 0.99). No statistically significant differences in breast cancer-specific mortality were observed for any racial or ethnic group. Cancer-specific mortality decreased following ACA implementation for colorectal and cervical cancers for some racial and ethnic groups in California, suggesting Medicaid expansion is associated with reductions in health inequity.

## Introduction

In the United States (U.S.), cancer care is inextricably linked with health insurance coverage (1). Prior studies have shown that, compared to privately-insured individuals, those who are uninsured or underinsured are less likely to undergo cancer screening and, therefore, more likely to present with later stage disease at time of diagnosis (2–4). Meanwhile, having health insurance is associated with shorter time to treatment initiation, receipt of guideline treatment, and improved survival (5).

The Patient Protection and Affordable Care Act (ACA), which was signed into law on March 23, 2010, and went into full effect January 1, 2014, expanded Medicaid eligibility to nonelderly adults with incomes at or below 138% of the federal poverty level with or without dependent children (6). To date, 39 U.S. states and the District of Columbia have adopted Medicaid expansion. Studies have shown that the ACA Medicaid expansion increased health insurance coverage among adults aged 18 to 64 years (7); moreover, this expansion has contributed to reductions in racial and ethnic disparities in coverage (8). Other provisions of the ACA are also relevant to cancer care. Starting in 2014, private health insurers could no longer deny coverage based on pre-existing conditions such as cancer, raise premiums, or deny coverage for cancer care. The ACA also created health insurance Marketplace plans, allowing the purchase of private health insurance plans, which are required to offer essential health benefits, including but not limited to hospitalizations, ambulatory care, and prescription drugs. The ACA’s required coverage for preventive care services, which includes screening for colon, breast, and cervical cancer without cost sharing (9), has begun to show benefits in increased cancer screening (10). These benefits of ACA implementation for cancer patients include reduced proportions of uninsured patients, earlier stage at diagnosis, improved care access, and decreased mortality (10–17). Despite variability in the implementation of Medicaid expansion, due to differences among state policies (18), few studies have reported cancer outcomes in individual states (19–21). Several studies have reported reductions in racial and ethnic disparities in early stage diagnosis (12, 22) but data on survival outcomes for different racial and ethnic groups are scarce (15, 23). Therefore, the true extent to which Medicaid expansion has resulted in specific improvements in cancer outcomes and reduced disparities among racial and ethnic populations remains unknown. Given the well-known racial and ethnic disparities in mortality that exist in the U.S. (24), understanding the extent to which the ACA implementation improves mortality and reduces disparities can help motivate additional initiatives to address mortality among particular racial or ethnic groups.

California was one of five U.S. states—including the District of Columbia—that opted to expand Medicaid coverage to its low-income residents before 2014 (25), and California now hosts the largest Medicaid program in the nation. This early Medicaid expansion in California, in concert with the state’s large and diverse population, provides a unique opportunity for assessing cancer outcomes in California following ACA implementation. As National Cancer Institute (NCI)-designated cancer centers in the U. S. charged with assessing and addressing the cancer burden in their catchment areas, results from this study provide important data for these centers in California to work with state and regional stakeholders to improve the health of the nearly 180,000 cancer patients who are diagnosed annually in the state and reduce the estimated 60,000 number of deaths that occur per year (26). Against this backdrop, we examined changes in the distributions of health insurance coverage, in the post- versus pre-ACA periods, among patients under 65 years of age in California with newly diagnosed breast, colorectal, and cervical cancer. In addition, we assessed cancer-specific mortality in the pre- and post-ACA periods for the three cancers, according to race and ethnicity. We limited our analysis to the three screenable cancers as these would benefit from ACA implementation through providing individuals with greater opportunities for early detection and, ultimately, reduced mortality. Breast cancer represents the second leading cause of cancer deaths in women and colorectal cancer is the third leading cause of cancer deaths in the United States (24). Even though cervix cancer is less common, mortality rates are higher in women of color than white women (24).

## Materials and Methods

### Study Participants and Disease Classification

Participants were identified among data retrieved from the California Cancer Registry (CCR), which is the largest population-based state cancer registry in the United States and contains demographic, diagnostic, treatment, and outcome information for cancer patients. The participant population comprised patients diagnosed in California aged less than 65 years with a first primary invasive female breast, colorectal, or cervical cancer between January 1, 2007 and December 31, 2017. We focused on this age group because newly diagnosed patients aged 65 years and older are age-eligible for Medicare coverage and the ACA provisions that affect insurance options affect people younger than age 65 years We used the International Classification of Diseases for Oncology, 3rd Edition (ICD-O-3) site codes C50.0–50.9 for breast cancer, C18.0–C18.9, C19.9, C20.9, and C26.0 for colorectal cancer, and C53.0–C53.9 for cervical cancer, excluding histology codes 9050–9055, 9140, and 9590–9992. Patients were excluded from analyses hierarchically as follows (See **Supplemental Figure 1**): diagnosis by death certificate or autopsy only (n = 878 breast; n = 1,231 colorectal; n = 77 cervical); not screening-eligible age (n = 98,966 not age 40–64 years for breast; n = 84,965 not age 50–64 years for colorectal; n = 2,604 not age 18–64 years for cervical); stage unknown (n = 4,638 for breast; n = 5,378 for colorectal; n = 863 for cervical); insurance status unknown (n = 2,952 for breast; n = 778 for colorectal; n = 296 for cervical); race and ethnicity other or unknown (n = 656 for breast; n = 255 for colorectal; n = 58 for cervical); no follow-up (n = 50 for breast; n = 38 for colorectal; n = 15 for cervical). The final study population included a total of 167,181 cancer patients: 117,738 breast, 38,334 colorectal, and 11,109 cervical cancer. Our study was approved by the institutional review boards at each of our respective institutions; informed consent was waived because we analyzed de-identified the data retrieved for analysis.

### Exposure Variables

Race and ethnicity were classified as American Indian/Alaska Native (AIAN), Asian American, Hispanic, Native Hawaiian/Pacific Islander (NHPI), non-Hispanic Black (NHB), or non-Hispanic White (NHW), according to patient medical records and CCR classification system, supplemented by the North American Association of Central Cancer Registries’ identification algorithm for Hispanic population groups using factors such as race and ethnicity, birthplace, and surnames. Primary and secondary sources of payment from the CCR data are based on the last admission for initial diagnosis and/or treatment. Because of the multiple ACA provisions that may affect access to cancer care outcomes, we included patients with all types of health insurance coverage. We classified insurance status according to five categories: private only; Medicare only or Medicare and private; any Medicaid; any military or public insurance other than Medicare or Medicaid; no insurance or self-pay. Given the increased recognition that the context within which we live, work, and play are important upstream determinants of health, we used a multi-component measure of neighborhood socioeconomic (nSES), based on patients’ residential census block group at diagnosis and American Community Survey data, categorized into quintiles based on the statewide distribution (27). We considered nSES as a potential confounder in the analysis, as it has been demonstrated in numerous studies to be independently associated with cancer outcomes, independent of individual or patient-level factors (28, 29).

### Follow-Up

Diagnosis year was categorized into three time periods: 2007–2010; 2011–2013; 2014–2017. We defined 2007–2010 as the period before the ACA implementation (i.e., pre-ACA) and 2014–2017 as the period after ACA implementation (i.e., post-ACA). Meanwhile, 2011–2013 was defined as the transition period before full Medicaid expansion, when individual counties in California were allowed to expand coverage and did so at different income thresholds (23). Follow-up time was calculated as the number of days from diagnosis until the date that occurred first among the following five: date of cancer-specific death (ICD-10 C50 for breast; C18, C19.9, C20.9, C26.0 for colorectal; C53 for cervical); date of death from another or unknown cause; date of last known contact; date 5 years after diagnosis; study end date of December 31, 2018. Follow-up time for survival extended through 2018 and was truncated at 5 years to allow for more equal opportunity for follow-up across the three time periods, and achieve balance between ensuring maximal inclusion of cases and contribution to follow up time. The median follow-up time for patients diagnosed in 2007–2010 was 5.0 years, and for 2011-2013 and 2014–2017, it was 5.0 years and 2.5 years, respectively.

### Statistical Analysis

Changes in insurance type were assessed by calculating the percent point difference between post-ACA (2014–2017) and pre-ACA (2007-2010) periods. For mortality analyses, the hazard rate ratio (HRR) and 95% confidence interval (CI) were calculated using multivariable Cox regression to estimate associations with risk of 5-year cancer-specific death. Patients with an unknown cause of death were excluded from the Cox analysis. The proportional hazards assumption was tested separately for each cancer site by examining the correlation between time and scaled Schoenfeld residuals for all variables. Variables that violated the proportional hazards assumption were included in the model as an underlying stratification factor, which allowed the baseline hazard to vary by the levels of these factors. Models were adjusted for clustering by Census block group using a sandwich estimator of the covariance structure that accounts for intracluster dependence. Breast cancer models included time-period, race and ethnicity, age, insurance status, marital status, tumor size, lymph node involvement, grade, histology, nSES, and diagnosis and/or treatment at an NCI cancer center; with underlying stratification by stage, breast cancer subtype as defined below, surgery, chemotherapy, and radiation; and clustering by block group. Breast cancer subtype was defined according to the following categories: hormone receptor (HR) positive (estrogen and/or progesterone receptor positive)/Her2neu (HER2) positive, HR+/HER2-, HR-/HER2+, and HR-/HER2-. Colorectal cancer models included time-period, race and ethnicity, age, sex, insurance status, marital status, tumor size, lymph node involvement, histology, anatomical subsite, surgery, nSES, and diagnosis and/or treatment at an NCI cancer center; with underlying stratification by stage, grade, chemotherapy, and radiation; and clustering by block group. Colorectal cancer models also were stratified by sex in secondary analysis; results were similar by sex. Cervical cancer models included time-period, race and ethnicity, age, insurance status, marital status, tumor size, lymph node involvement, grade, histology, surgery, nSES, and diagnosis and/or treatment at an NCI cancer center; with underlying stratification by stage, chemotherapy, and radiation; and clustering by block group. Wald global (and individual) tests for interaction were computed using cross-product terms in an overall, fully-adjusted model, which, to make the overall model comparable to the stratified models, featured underlying stratification by the stratification variable (race and ethnicity or time period) and was adjusted for all possible interactions with the stratification variable. Statistical significance was assessed with a threshold of p < 0.05. We conducted multivariable models to assess the intersectionality of ACA time period and race and ethnicity for each cancer site; one set of models assessed 5-year cancer-specific mortality by time period (2007–2010; 2011–2013; and 2014–2017) stratified by race and ethnicity and a second set assessed mortality by race and ethnicity stratified by time period. In addition, to assess potential changes resulting from the ACA implementation, we present two sets of multivariable models: one that does not include stage and treatment and one that does. All analyses were performed in SAS 9.4 (SAS Institute, Inc).

To assess the robustness of our Cox regression analysis, given that we do not include data from a non-Medicaid expansion region, we conducted a sensitivity analysis by including cancer patients aged 65 years and older as a comparison group, including 141,026 cancer patients (79,691 breast, 59,084 colorectal, and 2,251 cervical cancer) aged ≥65 years who met the same inclusion/exclusion criteria. A difference-in-difference analysis was used to compare mortality differences in post- vs. pre-ACA implementation between younger and older patients.

## Results

We included 117,738 breast, 38,334 colorectal, and 11,109 cervical cancer patients age <65 years who were newly diagnosed between 2007 and 2017 (**Table 1**). Differences in age distributions by cancer site reflect different screening eligibility ages for each cancer type, which resulted in a younger age for cervical cancer patients and an older age for colorectal cancer patients.

**Table 1:** Characteristics by Cancer Site in Patients Under 65 Years of Age, California, 2007–2017.

	Cancer Site					
	Female Breast		Colorectal Female & Male		Cervical	
	N	Col %	N	Col %	N	Col %
	117,738	100.0	38,334	100.0	11,109	100.0
**Age, Years**						
18–39	0	0.0	0	0.0	3,642	32.8
40–49	36,778	31.2	0	0.0	3,517	31.7
50–54	25,802	21.9	11,811	30.8	1,505	13.5
55–59	26,794	22.8	12,671	33.1	1,314	11.8
60–64	28,364	24.1	13,852	36.1	1,131	10.2
**Race and Ethnicity**						
AIAN	667	0.6	260	0.7	111	1.0
Asian	18,020	15.3	5,603	14.6	1,512	13.6
Hispanic	26,540	22.5	8,972	23.4	4,461	40.2
NHPI	739	0.6	215	0.6	90	0.8
NH Black	7,835	6.7	3,256	8.5	668	6.0
NH White	63,937	54.3	20,028	52.2	4,267	38.4
**Health Insurance**						
Private only	88,082	74.8	25,134	65.6	5,748	51.7
Medicare only or Medicare + Private	4,727	4.0	2,293	6.0	250	2.3
Any Medicaid	20,767	17.6	7,700	20.1	4,260	38.3
Any military/other public	2,866	2.4	1,966	5.1	477	4.3
No insurance	1,296	1.1	1,241	3.2	374	3.4
**Marital Status**						
Unmarried	40,812	34.7	14,676	38.3	5,634	50.7
Married	72,540	61.6	22,062	57.6	5,068	45.6
Unknown	4,386	3.7	1,596	4.2	407	3.7
**Year of Diagnosis**						
2007–2010	40,782	34.6	13,053	34.1	4,160	37.4
2011–2013	32,372	27.5	10,578	27.6	2,980	26.8
2014–2017	44,584	37.9	14,703	38.4	3,969	35.7
**AJCC Stage**						
I	53,955	45.8	10,070	26.3	5,822	52.4
II	42,669	36.2	8,299	21.6	1,237	11.1
III	15,105	12.8	11,137	29.1	2,464	22.2
IV	6,009	5.1	8,828	23.0	1,586	14.3
**Grade**						
Grade I	24,814	21.1	4,277	11.2	1,465	13.2
Grade II	48,463	41.2	23,961	62.5	3,381	30.4
Grade III/IV	39,106	33.2	5,809	15.2	3,373	30.4
Unknown	5,355	4.5	4,287	1.2	2,890	26.0
**Surgery**						
No	7,823	6.6	5,574	14.5	4,077	36.7
Yes	109,890	93.3	32,751	85.4	7,030	63.3
Unknown	25	0.0	9	0.0	<5	0.0
**Chemotherapy**						
No	57,033	48.4	18,522	48.3	5,775	52.0
Yes	58,794	49.9	18,934	49.4	5,205	46.9
Unknown	1,911	1.6	878	2.3	129	1.2
**Radiation**						
No	58,547	49.7	31,417	82.0	5,500	49.5
Yes	59,118	50.2	6,906	18.0	5,603	50.4
Unknown	73	0.1	11	0.0	6	0.1
**Neighborhood (Census Block Group) Socioeconomic Status Statewide Quintile**						
Quintile 1 (low)	14,876	12.6	6,340	16.5	2,812	25.3
Quintile 2	19,415	16.5	7,622	19.9	2,469	22.2
Quintile 3	22,586	19.2	7,878	20.6	2,201	19.8
Quintile 4	26,945	22.9	7,802	20.4	1,890	17.0
Quintile 5 (high)	30,397	25.8	7,355	19.2	1,428	12.9
Not geocodable	3,519	3.0	1,337	3.5	309	2.8
**Seen at an NCI-Designated Cancer Center**						
No	99,664	84.6	32,995	86.1	8,116	73.1
Yes	18,074	15.4	5,339	13.9	2,993	26.9

AIAN, American Indian or Alaska Native; NHPI, Native Hawaiian or Pacific Islander; NH, non-Hispanic.

We assessed changes in distributions of health insurance coverage in three separate time periods (2007–2010, 2011–2013, and 2014–2017) for breast, colorectal, and cervical cancer (**Table 2**). For breast cancer, after the ACA implementation, the percentage of uninsured women decreased by 0.3 percentage points, while the percentage of women with Medicaid increased by 2.4 percentage points, and those with private insurance decreased by 1.9 percentage points. Meanwhile, among patients with colorectal cancer, post-ACA, the proportion who were uninsured decreased by 2.3 percentage points, while those with Medicaid coverage increased by 9.5 percentage points, and those with privately insured patients decreased by 3.7 percentage points. Of the three cancers assessed, cervical cancer patients represented the highest proportion of uninsured patients pre-ACA (4.6%) and post-ACA (2.0%), exhibiting a 2.6 percentage-point decline. Medicaid coverage among cervical cancer patients increased by 1.4 percentage points, and private insurance increased by 2.3 percentage points. Changes in insurance coverage pre- and post-ACA, by racial and ethnic group, exhibited decreases in the proportion of uninsured patients for all groups, with some variation in the magnitude of the decrease across the groups (**Supplementary Table 1**). The proportion of Medicaid-insured individuals increased among Asian American, Hispanic, NHB, and NHW patients yet not among AIAN or NHPI patients. Private insurance decreased among NHB and NHW patients.

**Table 2:** Insurance Status by Time Period for Breast, Colorectal, and Cervical Cancer Patients Under 65 Years of Age, California, 2007–2017.

	Time Period						Difference^b^
	2007–2010		2011–2013		2014–2017		
	N	%	N	%	N	%	
**Breast Cancer**							
Insurance status^a^							
Private	31,245	76.6	23,536	72.7	33,301	74.7	-1.9
Medicare	1,622	4.0	1,370	4.2	1,735	3.9	-0.1
Medicaid	6,509	16.0	6,048	18.7	8,210	18.4	2.4
Other public	932	2.3	1,008	3.1	926	2.1	-0.2
Uninsured	474	1.2	410	1.3	412	0.9	-0.3
**Colorectal Cancer**							
Insurance status							
Private	8,922	68.4	6,698	63.3	9,514	64.7	-3.7
Medicare	795	6.1	686	6.5	812	5.5	-0.6
Medicaid	2,036	15.6	1,978	18.7	3,686	25.1	9.5
Other public	773	5.9	754	7.1	439	3.0	-2.9
Uninsured	527	4.0	462	4.4	252	1.7	-2.3
**Cervical Cancer**							
Insurance status							
Private	2,139	51.4	1,476	49.5	2,133	53.7	2.3
Medicare	80	1.9	71	2.4	99	2.5	0.6
Medicaid	1,543	37.1	1,188	39.9	1,529	38.5	1.4
Other public	205	4.9	145	4.9	127	3.2	-1.7
Uninsured	193	4.6	100	3.4	81	2.0	-2.6

a Insurance status categories: Private defined as private insurance only; Medicare defined as Medicare insurance only or Medicare and private insurance; Medicaid defined as any Medicaid insurance; Other public defined as any public insurance other than Medicare and Medicaid; Uninsured defined as no health insurance.

b Difference between 2014–2017 and 2007–2010 time periods.

**Table 3** shows 5-year cancer-specific mortality, by time period, for breast, colorectal and cervical cancer, stratified by race and ethnicity, for the four largest racial and ethnic groups. No significant differences in mortality were observed over time for breast cancer patients. In the fully-adjusted model, risk of dying from colorectal cancer was significantly lower in the post- vs. pre-ACA periods for Hispanic (HRR = 0.82, 95% CI = 0.74 to 0.92), NHB (HRR = 0.69, 95% CI = 0.58 to 0.82), and NHW (HRR = 0.90, 95% CI = 0.84 to 0.97) patients but not Asian American (HRR = 0.95, 95% CI = 0.82 to 1.10) patients. A statistically significant interaction between race and ethnicity and time period was observed in the fully-adjusted model (global p-interaction = 0.033); specifically, the HRR for NHB cases in 2011-2013 and 2014-2017 (compared to 2007-2010) were statistically significantly lower than the HRR for NHW cases (individual p-interaction = 0.005 for both time-periods). In addition, a statistically significantly lower risk of dying from cervical cancer was observed in the post- vs. pre-ACA period among NHB women in the fully adjusted model (HRR = 0.68, 95% CI = 0.47 to 0.99). When we assessed cancer-specific mortality differences by race and ethnicity stratified by time period, NHB women had a higher risk of dying from breast cancer compared to NHW patients for all three time periods (**Supplemental Table 2**). Whereas for colorectal cancer, NHB cases had a higher risk of dying from colorectal cancer compared to NHW cases only in the pre-ACA time-period.

**Table 3:** Risk of 5-year cancer-specific death for time-period stratified by race/ethnicity among breast, colorectal, and cervix cancer patients less than 65 years of age, California, 2007-2017.

	NH White		Asian American		Hispanic		NH Black		p-int^a,c^	p-int^b,c^
	HRR^a^(95% CI)	HRR^b^(95% CI)	HRR^a^(95% CI)	HRR^b^(95% CI)	HRR^a^(95% CI)	HRR^b^(95% CI)	HRR^a^(95% CI)	HRR^b^(95% CI)		
**Breast cancer**										
Time-period										
2007-2010	1.00	1.00	1.00	1.00	1.00	1.00	1.00	1.00	0.061	0.141
2011-2013	0.94 (0.87-1.01)	0.94 (0.87-1.01)	0.96 (0.82-1.14)	0.95 (0.80-1.12)	0.98 (0.88-1.10)	0.95 (0.85-1.05)	1.13^d^ (0.99-1.30)	1.07 (0.93-1.23)		
2014-2017	1.02 (0.94-1.11)	0.97 (0.89-1.05)	1.16 (0.98-1.38)	1.10 (0.92-1.31)	1.00 (0.89-1.11)	0.92 (0.82-1.04)	0.97 (0.83-1.13)	0.88 (0.75-1.04)		
**Colorectal cancer**										
Time-period										
2007-2010	1.00	1.00	1.00	1.00	1.00	1.00	1.00	1.00	0.455	**0.033**
2011-2013	**1.09** **(1.02-1.17)**	1.03 (0.96-1.11)	1.07 (0.93-1.23)	1.03 (0.90-1.19)	1.07 (0.96-1.18)	0.97 (0.87-1.08)	0.97 (0.83-1.13)	**0.81** ^d^ **(0.70-0.95)**		
2014-2017	1.03 (0.95-1.10)	**0.90** **(0.84-0.97)**	1.09 (0.94-1.26)	0.95 (0.82-1.10)	0.94 (0.84-1.04)	**0.82** **(0.74-0.92)**	0.93 (0.79-1.10)	**0.69** ^d^ **(0.58-0.82)**		
**Cervix cancer**										
Time-period										
2007-2010	1.00	1.00	1.00	1.00	1.00	1.00	1.00	1.00	0.543	0.257
2011-2013	0.98 (0.83-1.17)	0.93 (0.79-1.10)	1.05 (0.76-1.43)	1.14 (0.83-1.58)	1.08 (0.91-1.28)	1.10 (0.93-1.30)	0.93 (0.65-1.32)	1.04 (0.72-1.49)		
2014-2017	1.09 (0.92-1.29)	1.00 (0.85-1.18)	1.09 (0.79-1.49)	1.09 (0.80-1.49)	1.07 (0.90-1.27)	1.03 (0.86-1.22)	0.73 (0.51-1.05)	**0.68** **(0.47-0.99)**		

NH, non-Hispanic; HR, hazard rate ratio; CI, confidence interval; nSES, neighborhood socioeconomic status; NCI, National Cancer Institute.

a Cox regression. Breast cancer: Model adjusted for age, insurance status, marital status, tumor size, lymph node involvement, grade, histology, nSES, and NCI cancer center; with underlying stratification by HR/HER2 subtype; and clustering by block group. Colorectal cancer: Model adjusted for age, sex, insurance status, marital status, tumor size, lymph node involvement, histology, anatomical subsite, nSES, and NCI cancer center; with underlying stratification by grade; and clustering by block group. Cervical cancer: Model adjusted for age, insurance status, marital status, tumor size, lymph node involvement, grade, histology, nSES, and NCI cancer center; and clustering by block group.

b Cox regression. Breast cancer: Same as model in footnote a with additional underlying stratification by stage, surgery, chemotherapy, and radiation. Colorectal cancer: Same as model in footnote a additionally adjusted for surgery; with additional underlying stratification by stage, chemotherapy, and radiation. Cervical cancer: Same as model in footnote a additionally adjusted for surgery; with additional underlying stratification by stage, chemotherapy, and radiation.

c Global p-interaction in a fully adjusted overall model with underlying stratification by race/ethnicity and including all possible cross-product interactions with race/ethnicity.

d Significantly different from NH White (individual cross-product interaction term P<0.05 in a fully adjusted overall model with underlying stratification by race/ethnicity and including all possible cross-product interactions with race/ethnicity). Bold text indicates statistical significance.

**Figure 1:**
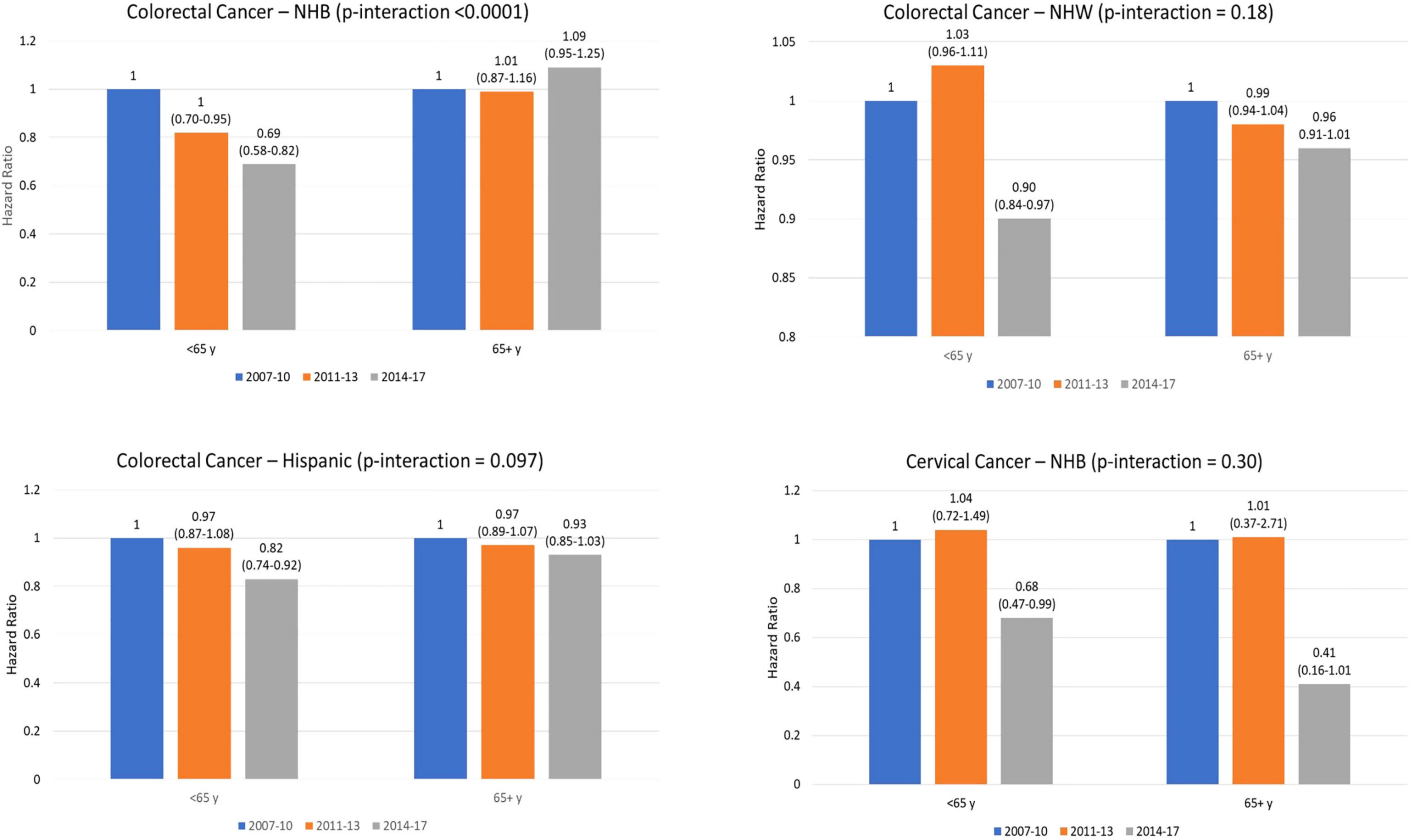
Hazard Ratio (95% coinfedence interval) for cancer-specific death by time period for younger (<65 years) and older (65 years and older) patienets. P-value indicates interaction for difference in mortality between 2014-2017 compared to 2007-2010 between older and younger patients. Note. NHB, non-Hispanic Black; NHW. Non-Hispanic white.

In a sensitivity analysis, we assessed 5-year cancer-specific mortality by time period for breast, colorectal, and cervical cancer, stratified by race and ethnicity, for cancer patients aged 65 years and older (**Table 4**). Contrary to the mortality differences observed among younger patients in the fully-adjusted models, no statistically significant differences were observed for the post- vs. pre-ACA period for any racial and ethnic group among patients ≥65 years with breast, colorectal, or cervical cancer. **Figure 1** shows results of the difference-in-difference analysis for the associations found to be statistically significant in the younger group. A significantly lower mortality post- vs pre-ACA was observed in younger compared to older NHB colorectal cancer patients (p-interaction < 0.0001).

**Table 4:** Risk of 5-year Cancer-Specific Death for Time Period Stratified by Race and Ethnicity among Breast, Colorectal, and Cervical Cancer Patients 65 Years of Age or Older, California, 2007–2017.

	NH White		Asian American		Hispanic		NH Black		pa
	HRR	(95% CI)	HRR	(95% CI)	HRR	(95% CI)	HRR	(95% CI)	
Breast Cancer^b^									
Time period									
2007–2010	1.00		1.00		1.00		1.00		0.696
2011–2013	1.00	(0.93 to 1.07)	0.99	(0.80 to 1.23)	0.92	(0.79 to 1.08)	0.84	(0.68 to 1.04)	
2014–2017	0.97	(0.90 to 1.05)	1.05	(0.84 to 1.31)	0.89	(0.76 to 1.05)	0.95	(0.77 to 1.18)	
Colorectal Cancer^c^									
Time period									
2007–2010	1.00		1.00		1.00		1.00		0.660
2011–2013	0.99	(0.94 to 1.04)	0.96	(0.86 to 1.07)	0.97	(0.89 to 1.07)	1.01	(0.87 to 1.16)	
2014–2017	0.96	(0.91 to 1.01)	0.94	(0.84 to 1.05)	0.93	(0.85 to 1.03)	1.09	(0.95 to 1.25)	
Cervical Cancer^d^									
Time period									
2007–2010	1.00		1.00		1.00		1.00		0.171
2011–2013	1.35	(1.00 to 1.83)	2.13	(1.11 to 4.09)	1.02	(0.68 to 1.52)	1.01	(0.37 to 2.71)	
2014–2017	1.00	(0.75 to 1.33)	1.79	(0.92 to 3.49)	1.05	(0.70 to 1.56)	0.41	(0.16 to 1.01)	

Bold text indicates statistical significance.

a Global p-interaction in a fully adjusted overall model with underlying stratification by race and ethnicity and including all possible cross-product interactions with race and ethnicity.

b Breast cancer model adjusted for age, insurance status, marital status, tumor size, lymph node involvement, grade, histology, nSES, and NCI cancer center; with underlying stratification by stage, HR/HER2 subtype, surgery, chemotherapy, and radiation; and clustering by block group.

c Colorectal cancer model adjusted for age, sex, insurance status, marital status, tumor size, lymph node involvement, histology, anatomical subsite, surgery, nSES, and NCI cancer center; with underlying stratification by stage, grade, chemotherapy, and radiation; and clustering by block group.

d Cervical cancer model adjusted for age, insurance status, marital status, tumor size, lymph node involvement, grade, histology, surgery, nSES, and NCI cancer center; with underlying stratification by stage, chemotherapy, and radiation; and clustering by block group.

Given the small number of AIAN and NHPI patients represented in the CCR, we were unable to assess differences over time period stratified by these two racial groups. Nevertheless, in separate analyses to assess 5-year cancer-specific mortality for race and ethnicity stratified by time period (**Supplementary Table 2**), we observed higher (not statistically significant) breast cancer mortality among NHPI patients compared to NHW patients in the three time periods, yet we observed lower mortality for Asian American women. Similar mortality differences between NHPI and Asian American patients compared to NHW patients were observed for colorectal and cervical cancer. A mortality difference across time for AIAN patients compared to NHW patients was observed for cervical cancer: a lower yet non-statistically significant risk of dying was shown in the post-ACA period (HRR = 0.48, 95% CI = 0.20 to 1.17), which was not present before ACA implementation (HRR = 1.17; 95% CI = 0.70 to 1.97).

## Discussion

We assessed changes in distributions of health insurance coverage and 5-year cancer-specific mortality following implementation of the ACA among breast, colorectal, and cervical cancer patients younger than age 65 years, in California, an early adopter of Medicaid expansion under the ACA and the most populous U.S. state. Although reports on the effect of this landmark legislation in the U.S. have been published (7–12, 22), data on cancer-specific mortality, particularly by racial and ethnic group, are scarce. In addition to decreases in the proportion of uninsured patients for all three cancers, across all racial and ethnic groups, we observed a decline in cancer-specific mortality following full ACA implementation. These results show the benefits of ACA implementation among NHB and Hispanic colorectal cancer patients and among NHB cervical cancer patients.

One goal of the ACA is to improve patient outcomes, which includes cancer survival. Published data on mortality outcomes for breast and colorectal cancer related to the ACA are mixed and scarce (15, 20, 23). To date, most studies have reported benefits of the ACA on cancer screening and stage at diagnosis (11, 14, 30). According to our research, there are two published reports on cancer mortality changes following ACA implementation, stratified by race and ethnicity (15, 23). One study reported no differences in breast, colorectal, and lung cancer mortality combined, between Black and White patients (15). In the second study (23), a two-year survival benefit associated with Medicaid expansion was greater in NHB patients, which resulted in narrowing disparities in cancer survival, similar to our findings. Our study addresses the limited literature on cancer-specific mortality (15, 20) and extends our understanding of racial and ethnic differences. We observed post-ACA reductions in colorectal cancer mortality of 10% among NHW, 18% among Hispanic, and 31% among NHB patients under age 65 years. Though, no significant changes in mortality were shown following the ACA for patients 65 years and older. These findings are consistent with a report from Kentucky (20) and a large population-based national study (23) yet inconsistent with those reported by Lam et al., (15). Contrary to reported findings (15), we observed no differences in breast cancer mortality when comparing the post- and pre-ACA periods. This could be because (1) the percent of uninsured breast cancer patients in California pre-ACA was very low and (2) the reduction in uninsured women post-ACA was small. For patients with cervical cancer, NHB women exhibited a statistically significant, lower mortality post-ACA compared to pre-ACA, but this was not observed for other racial and ethnic groups. This lack of association could be due to insufficient power to detect differences, given it is a less common malignancy compared to the others. This could be because cervical cancer patients exhibited the smallest gains in Medicaid coverage and had the highest rate of being uninsured following ACA implementation. In addition, for breast and cervical cancer outcomes, the effect of the ACA could be weakened by that of existing screening programs in California for uninsured and low-income women, which has been described in the literature (7, 8). Although our findings suggest a narrowing in some of the racial and ethnic disparities following ACA implementation, we were unable to conduct analyses specific to AIAN and NHPI patients, due to their small representation in the CCR.

Reports on changes in proportions of uninsured cancer patients following ACA implementation show greater reductions in Medicaid expansion states compared to non-expansion states (11, 14, 30–33). Among expansion states, a 2.6–2.9 percentage point drop in the proportion of uninsured cancer patients has been shown for post- vs. pre-ACA periods (11, 14, 26), which is consistent with our results for California. In addition, our results show that improvements in insurance coverage occurred among all racial and ethnic groups, which range from a 0.6 percentage point decrease in the proportion uninsured for NHW patients to a 1.6 decrease for Hispanic patients. Indeed, Han et al. (26), also have reported these higher decreases among Hispanic patients compared to other racial and ethnic groups. Meanwhile, breast cancer patients exhibited the smallest decrease (0.3%) in being uninsured; however, these patients began with low uninsured rates prior to ACA implementation (1.2%). Larger decreases in the proportion of uninsured individuals were observed for colorectal cancer patients (2.3 percentage points), which could be due to larger reductions in uninsured male compared to female patients, as reported in the literature (12). Although cervical cancer patients exhibited the highest decrease in being uninsured (2.6 percentage points) for all three cancers, these patients also exhibited the highest proportion of uninsured women pre- and post-ACA implementation. These results underscore the challenges faced by cervical cancer patients, who are younger than colorectal and breast cancer patients and, as such, may experience unstable coverage. Our study contributes to and expands the literature through assessing corresponding changes in Medicaid coverage following ACA implementation. Colorectal cancer patients exhibited the largest gains in Medicaid coverage (9.5 percentage points), whereas cervical cancer patients exhibited the lowest increase (1.4 percentage points), changes that are within the range of those reported in the literature (11, 14).

Our study of California exhibits several strengths: the population-based nature of its data; the racial and ethnic diversity of its participants; the relatively long follow-up post-ACA because California was an early-Medicaid expansion state. Nevertheless, with these strengths come some limitations. First, this study is representative of California, which may limit its generalizability to other U.S. states. Indeed, prior studies that have analyzed multiple states comprise a mix of early- and late-Medicaid expansion regions, which feature differences in baseline mortality (15); this mix may obscure results, as has been reported (14). Further, we did not include a non-expansion state for comparison, as others have done. Instead, we compared outcomes to cancer patients 65 years of age and older, who were not affected by the coverage expansion provisions of the ACA we are examining in this study. Second, unmeasured confounders also could have contributed to our observation of decreasing cancer rates, as demonstrated by the down-trending rates in the pre-ACA era. Although we had the advantage of longer follow-up post-ACA, we still were limited by the relatively small number of outcomes for cervical cancer mortality, which resulted in imprecise measures of association, and the relatively small number of AIAN and NHPI cases. Third, because health insurance status, as recorded in CCR data, is based on last admission for initial diagnosis and treatment. We were unable to ascertain health insurance changes following cancer diagnosis or treatment.

In conclusion, following ACA implementation in California, a decrease in the proportion of uninsured patients was observed among non-elderly, newly diagnosed breast, colorectal, and cervical cancer patients, which varied by cancer site and by racial and ethnic group. In addition, lower cancer-specific mortality was observed for NHW, NHB, and Hispanic colorectal cancer patients, and for NHB cervical cancer patients in the post- vs. pre-ACA phase. These results contribute to ongoing discussions regarding healthcare reform in the United States, as additional states consider Medicaid expansion against the backdrop of further efforts to weaken the ACA. Moreover, given the economic impact of the COVID-19 pandemic, which has resulted in unemployment and lost health coverage, future analyses should assess shifts between health insurance coverage plans on cancer outcomes. Doing so could contribute to expanding Medicaid in particular U.S. states to address health inequity.

## Data Availability Statement

Publicly available datasets were analyzed in this study. This data can be found here: California Cancer Registry: ccrcal.org.

## Author Contributions

Conceptualization: JM, SLG, DO, KRY, MM. Formal Analysis: AC. Funding acquisition: SLG, MM. Investigation: JM, SLG, MM. Methodology: JM, SLG, DO, AC, MB, MM. Resources: SLG. Software: SLG. Supervision: SLG, MM. Writing – original draft: AC, MB, MM. Writing – review & editing: JM, SLG, DO, AC, WM, RY, MB, MM.

## Funding

This work was supported by the National Cancer Institute at the National Institutes of Health (grant numbers U54CA132379, U54CA132384, 5P30CA023100, UH3CA233314). The collection of cancer incidence data used in this study was supported by the California Department of Public Health pursuant to California Health and Safety Code Section 103885; Centers for Disease Control and Prevention’s (CDC) National Program of Cancer Registries, under cooperative agreement 5NU58DP006344; the National Cancer Institute’s Surveillance, Epidemiology and End Results Program under contract HHSN261201800032I awarded to the University of California, San Francisco, contract HHSN261201800015I awarded to the University of Southern California, and contract HHSN261201800009I awarded to the Public Health Institute, Cancer Registry of Greater California. The ideas and opinions expressed herein are those of the author(s) and do not necessarily reflect the opinions of the State of California, Department of Public Health, the National Cancer Institute, and the Centers for Disease Control and Prevention or their Contractors and Subcontractors.

## Conflict of Interest

WM: Flatiron Health - honorarium for a talk in disparities in healthcare. KRY: Serves on the Flatiron Health Equity Advisory Board. All honoraria are donated to her employer, the American Cancer Society.

The remaining authors declare that the research was conducted in the absence of any commercial or financial relationships that could be construed as a potential conflict of interest.

## Publisher’s Note

All claims expressed in this article are solely those of the authors and do not necessarily represent those of their affiliated organizations, or those of the publisher, the editors and the reviewers. Any product that may be evaluated in this article, or claim that may be made by its manufacturer, is not guaranteed or endorsed by the publisher.
