# Supplementary material for: Changes in Cancer Mortality by Race and Ethnicity Following the Implementation of the Affordable Care Act in California
Source: Front Oncol. 2022 Jul 13;12:916167. doi: 10.3389/fonc.2022.916167 (PMC9327742; doi:10.3389/fonc.2022.916167)
Supplement: Supplementary file 1 [file DataSheet_1.docx]

Supplementary Material


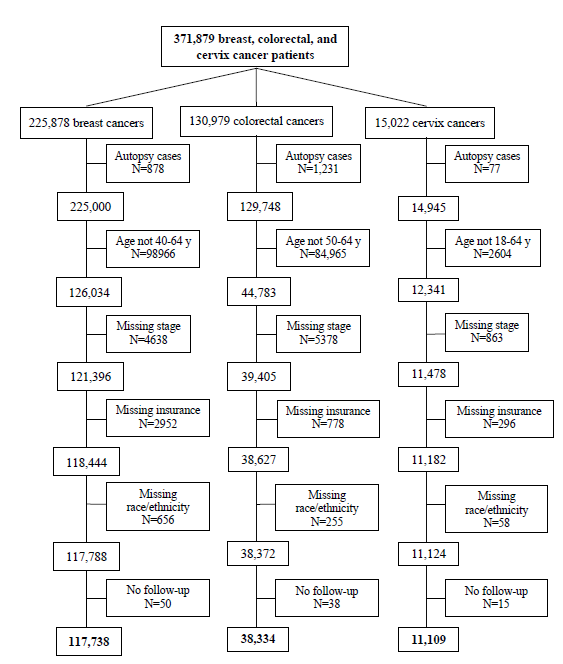


**Supplemental Figure 1. Flow chart of exclusion criteria applied to derive at the final study population**

| **Supplementary Table 1. Insurance Status by Race and Ethnicity for Breast, Colorectal and Cervical Cancer Patients Under 65 Years of Age, California, 2007–2017** | | | | | | | |
| --- | --- | --- | --- | --- | --- | --- | --- |
|  | **Time Period** | | | | | | **Difference**^b^ |
|  | 2007–2010 | | 2011–2013 | | 2014–2017 | |  |
|  | N | % | N | % | N | % |  |
| **AIAN** |  |  |  |  |  |  |  |
| Insurance status^a^ |  |  |  |  |  |  |  |
| Private | 192 | 58.5 | 142 | 51.3 | 287 | 66.3 | 7.8 |
| Medicare | 22 | 6.7 | 19 | 6.9 | 26 | 6.0 | -0.7 |
| Medicaid | 89 | 27.1 | 100 | 36.1 | 105 | 24.2 | -2.9 |
| Other public | 20 | 6.1 | 14 | 5.1 | 14 | 3.2 | -2.9 |
| Uninsured | 5 | 1.5 | < 5 |  | < 5 |  |  |
| **Asian American** |  |  |  |  |  |  |  |
| Insurance status |  |  |  |  |  |  |  |
| Private | 5,868 | 73.2 | 4,742 | 69.8 | 7,694 | 74.5 | 1.3 |
| Medicare | 220 | 2.7 | 213 | 3.1 | 252 | 2.4 | -0.3 |
| Medicaid | 1,401 | 17.5 | 1,342 | 19.8 | 1,964 | 19.0 | 1.5 |
| Other public | 337 | 4.2 | 362 | 5.3 | 296 | 2.9 | -1.3 |
| Uninsured | 185 | 2.3 | 135 | 2.0 | 124 | 1.2 | -1.1 |
| **Hispanic** |  |  |  |  |  |  |  |
| Insurance status |  |  |  |  |  |  |  |
| Private | 7,002 | 56.4 | 5,924 | 53.8 | 9,371 | 56.6 | 0.2 |
| Medicare | 449 | 3.6 | 444 | 4.0 | 621 | 3.8 | 0.2 |
| Medicaid | 4,106 | 33.1 | 3,726 | 33.9 | 5,858 | 35.4 | 2.3 |
| Other public | 439 | 3.5 | 545 | 5.0 | 414 | 2.5 | -1.0 |
| Uninsured | 420 | 3.4 | 363 | 3.3 | 291 | 1.8 | -1.6 |
| **NHPI** |  |  |  |  |  |  |  |
| Insurance status |  |  |  |  |  |  |  |
| Private | 194 | 67.4 | 204 | 67.8 | 322 | 70.8 | 3.4 |
| Medicare | 10 | 3.5 | 12 | 4.0 | 21 | 4.6 | 1.1 |
| Medicaid | 60 | 20.8 | 62 | 20.6 | 89 | 19.6 | -1.2 |
| Other public | 17 | 5.9 | 17 | 5.6 | 19 | 4.2 | -1.7 |
| Uninsured | 7 | 2.4 | 6 | 2.0 | < 5 |  |  |
| **NH Black** |  |  |  |  |  |  |  |
| Insurance status |  |  |  |  |  |  |  |
| Private | 2,635 | 62.7 | 1,971 | 58.3 | 2,593 | 62.1 | -0.6 |
| Medicare | 216 | 5.1 | 209 | 6.2 | 212 | 5.1 | 0.0 |
| Medicaid | 1,030 | 24.5 | 918 | 27.1 | 1,167 | 28.0 | 3.5 |
| Other public | 222 | 5.3 | 196 | 5.8 | 147 | 3.5 | -1.8 |
| Uninsured | 101 | 2.4 | 88 | 2.6 | 54 | 1.3 | -1.1 |
| **NH White** |  |  |  |  |  |  |  |
| Insurance status |  |  |  |  |  |  |  |
| Private | 26,415 | 80.7 | 18,727 | 77.5 | 24,681 | 78.8 | -1.9 |
| Medicare | 1,580 | 4.8 | 1,230 | 5.1 | 1,514 | 4.8 | 0.0 |
| Medicaid | 3,402 | 10.4 | 3,066 | 12.7 | 4,242 | 13.5 | 3.1 |
| Other public | 875 | 2.7 | 773 | 3.2 | 602 | 1.9 | -0.8 |
| Uninsured | 476 | 1.5 | 378 | 1.6 | 271 | 0.9 | -0.6 |
| *Note*. AIAN = American Indian or Alaska Native; NHPI = Native Hawaiian or Pacific Islander; NH = Non-Hispanic.  ^a^Insurance status categories: Private defined as private insurance only; Medicare defined as Medicare insurance only or Medicare and private insurance; Medicaid defined as any Medicaid insurance; Other public defined as any public insurance other than Medicare and Medicaid; Uninsured defined as no health insurance.  ^b^Difference between 2014–2017 and 2007–2010 time periods. | | | | | | | |

| **Supplementary Table 2. Risk of 5-Year Cancer-Specific Death for Race and Ethnicity, Stratified by Time-Period among Breast, Colorectal, and Cervical Cancer Patients Under 65 Years of Age, California, 2007–2017** | | | | | | | |  | |
| --- | --- | --- | --- | --- | --- | --- | --- | --- | --- |
|  | **Time Period** | | | | | |  |  | |
|  | 2007–2010 | | 2011–2013 | | 2014–2017 | |  |  | |
|  | HR | (95% CI) | HR | (95% CI) | HR | (95% CI) | p^a^ |  | |
| **Breast Cancer**^b^ |  |  |  |  |  |  |  |  | |
| Race and ethnicity |  |  |  |  |  |  |  | |  |
| NH White | 1.00 |  | 1.00 |  | 1.00 |  | 0.561 |  | |
| AIAN | 1.41 | (0.95 to 2.09) | 0.90 | (0.52 to 1.55) | 1.35 | (0.80 to 2.28) |  |  | |
| Asian American | **0.80** | **(0.71 to 0.90)** | **0.81** | **(0.71 to 0.92)** | 0.88 | (0.77 to 1.01) |  |  | |
| Hispanic | 0.91 | (0.83 to 1.00) | **0.88** | **(0.79 to 0.98)** | 0.89 | (0.79 to 1.00) |  |  | |
| NHPI | 1.15 | (0.75 to 1.77) | 1.34 | (0.87 to 2.06) | 1.18 | (0.78 to 1.79) |  |  | |
| NH Black | **1.27** | **(1.13 to 1.42)** | **1.40** | **(1.23 to 1.58)** | **1.16** | **(1.00 to 1.35)** |  |  | |
| **Colorectal Cancer**^c^ |  |  |  |  |  |  |  |  | |
| Race and ethnicity |  |  |  |  |  |  |  |  | |
| NH White | 1.00 |  | 1.00 |  | 1.00 |  | 0.101 |  | |
| AIAN | 0.86 | (0.54 to 1.38) | 1.01 | (0.60 to 1.69) | 1.09 | (0.72 to 1.65) |  |  | |
| Asian American | 0.93 | (0.84 to 1.03) | 0.95 | (0.84 to 1.07) | 0.99 | (0.88 to 1.11) |  |  | |
| Hispanic | 0.97 | (0.88 to 1.07) | **0.90** | **(0.82 to 0.99)** | **0.89** | **(0.81 to 0.98)** |  |  | |
| NHPI | 0.85 | (0.58 to 1.24) | 1.07 | (0.68 to 1.67) | 1.40 | (0.88 to 2.24) |  |  | |
| NH Black | **1.20** | **(1.07 to 1.34)** | 0.95^e^ | (0.83 to 1.08) | 0.94^e^ | (0.81 to 1.08) |  |  | |
| **Cervical Cancer**^d^ |  |  |  |  |  |  |  |  | |
| Race and ethnicity |  |  |  |  |  |  |  |  | |
| NH White | 1.00 |  | 1.00 |  | 1.00 |  | 0.448 |  | |
| AIAN | 1.17 | (0.70 to 1.97) | 1.09 | (0.59 to 2.03) | 0.48 | (0.20 to 1.17) |  |  | |
| Asian American | 0.80 | (0.64 to 1.00) | 0.95 | (0.73 to 1.24) | 0.84 | (0.64 to 1.08) |  |  | |
| Hispanic | **0.76** | **(0.64 to 0.90)** | 0.95 | (0.78 to 1.15) | 0.84 | (0.70 to 1.01) |  |  | |
| NHPI | 1.70 | (0.92 to 3.13) | 1.85 | (0.96 to 3.57) | 1.51 | (0.89 to 2.56) |  |  | |
| NH Black | 1.22 | (0.94 to 1.56) | **1.43** | **(1.08 to 1.89)** | 0.96 | (0.72 to 1.28) |  |  | |
| *Note*. HR = hazard rate ratio; CI = confidence interval; NH = non-Hispanic; AIAN = American Indian or Alaska Native; NHPI = Native Hawaiian or Pacific Islander; nSES = neighborhood socioeconomic status; NCI = National Cancer Institute; Bold text indicates statistical significance.  ^a^Global p-interaction in a fully adjusted overall model with underlying stratification by time-period and including all possible cross-product interactions with time-period.  ^b^Breast cancer model adjusted for age, insurance status, marital status, tumor size, lymph node involvement, grade, histology, nSES, and NCI cancer center; with underlying stratification by stage, HR/HER2 subtype, surgery, chemotherapy, and radiation; and clustering by block group.  ^c^Colorectal cancer model adjusted for age, sex, insurance status, marital status, tumor size, lymph node involvement, histology, anatomical subsite, surgery, nSES, and NCI cancer center; with underlying stratification by stage, grade, chemotherapy, and radiation; and clustering by block group.  ^d^Cervical cancer model adjusted for age, insurance status, marital status, tumor size, lymph node involvement, grade, histology, surgery, nSES, and NCI cancer center; with underlying stratification by stage, chemotherapy, and radiation; and clustering by block group.  ^e^Statistically significant difference from 2007–2010 (individual cross-product interaction term p < 0.05 in a fully adjusted overall model, with underlying stratification by time-period and including all possible cross-product interactions with time-period). | | | | | | | |  | |
